# MFF-Net: Deepfake Detection Network Based on Multi-Feature Fusion

**DOI:** 10.3390/e23121692

**Published:** 2021-12-17

**Authors:** Lei Zhao, Mingcheng Zhang, Hongwei Ding, Xiaohui Cui

**Affiliations:** Key Laboratory of Aerospace Information Security and Trusted Computing, Ministry of Education, School of Cyber Science and Engineering, Wuhan University, Wuhan 430072, China; zhao_lei@whu.edu.cn (L.Z.); mczhang@whu.edu.cn (M.Z.); hwding@whu.edu.cn (H.D.)

**Keywords:** deepfake, feature fusion, attention, generative adversarial network

## Abstract

Significant progress has been made in generating counterfeit images and videos. Forged videos generated by deepfaking have been widely spread and have caused severe societal impacts, which stir up public concern about automatic deepfake detection technology. Recently, many deepfake detection methods based on forged features have been proposed. Among the popular forged features, textural features are widely used. However, most of the current texture-based detection methods extract textures directly from RGB images, ignoring the mature spectral analysis methods. Therefore, this research proposes a deepfake detection network fusing RGB features and textural information extracted by neural networks and signal processing methods, namely, MFF-Net. Specifically, it consists of four key components: (1) a feature extraction module to further extract textural and frequency information using the Gabor convolution and residual attention blocks; (2) a texture enhancement module to zoom into the subtle textural features in shallow layers; (3) an attention module to force the classifier to focus on the forged part; (4) two instances of feature fusion to firstly fuse textural features from the shallow RGB branch and feature extraction module and then to fuse the textural features and semantic information. Moreover, we further introduce a new diversity loss to force the feature extraction module to learn features of different scales and directions. The experimental results show that MFF-Net has excellent generalization and has achieved state-of-the-art performance on various deepfake datasets.

## 1. Introduction

**Figure 1:**
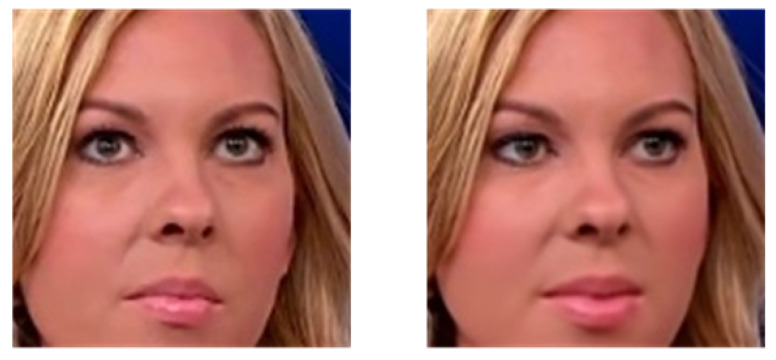
Examples of a real face and fake face generated by NeuralTextures. **Left**: natural face from FaceForensics++ [8]. **Right**: fake face synthesized by NeuralTextures [9]. Forged images and authentic images are indistinguishable to the human eye.

Artificial intelligence has transformed all aspects of life, including facial recognition, fast identity authentication, logging into mobile apps, and making payments. However, the development of deep-learning-driven forged image generation models [1,2,3,4] allows attackers to create realistic facial images, as shown in Figure 1. Most of them cannot be distinguished by human eyes. A survey by whichfaceisreal.com [5] shows that users cannot distinguish between real and fake images well. According to this whichfaceisreal.com survey, although it is generally believed that human eye recognition is better than random guessing, users only achieve a maximum recognition accuracy of 75%. There are many mature tools for generating fake images and videos, such as FaceApp [6] and FaceSwap [7]. Even ordinary users can use tools to generate fake videos without understanding generative adversarial networks’ principles and can spread them on the internet or even make profits.

Extensive and excellent work on deepfake detection has been carried out to deal with the deepfake challenge [8,10,11,12,13]. At present, most advanced detection methods are based on RGB images. A detection method based on RGB images [10,14] can achieve a higher accuracy rate on datasets generated by a single generative adversarial network (GAN). However, this method may be influenced greatly by the structures of generating networks and dataset diversity. Only images and videos generated by a specific generation method can be recognized, and the results of testing on new datasets will be terrible. To address this problem, researchers have begun to focus on general forged features [15,16], such as texture and frequency spectra.

GANs are widely used in deepfake generation technology. A GAN learns the distribution of real samples and gradually increases the dimension of random low-dimensional space to generate forged samples. However, due to the limited receptive field of the generated network, a GAN cannot learn the global textural information, resulting in an immediate difference between real samples and forged samples. Many methods based on textural defects have been proposed [17]. However, most of the current texture-based detection methods extract textures directly from RGB images, ignoring the mature signal processing methods. In addition, upsampling is widely used in the generators of GANs, but it causes spectral defects, such as in the grid structure of a spectrum or an abnormal high-frequency part [15], which is the principle of frequency-based detection methods. Since a neural network can not directly obtain frequency features from RGB images, most of the existing studies [18,19] used the discrete Fourier transform to process RGB images to obtain the spectra. However, frequency-based methods lose semantic information in extracting frequency domain features. In addition, traditional frequency domain methods, such as fast Fourier transform and discrete cosine transform, mismatch the translation invariance and local consistency of natural images. Therefore, a traditional CNN is not suitable for this method.

In order to make better use of textural and frequency features, Gabor convolution was designed with reference to the Gabor filter to extract information in different directions and scales. The Gabor transform is a special case of a short-time-windowed Fourier transform when the window function is a Gaussian function. Therefore, the Gabor filter can extract relevant features in different scales and directions in the frequency domain. In addition, the Gabor function is similar to the function of the human eye, so it is often used in texture recognition and has achieved good results.

We propose a new feature fusion network for deepfake detection. First, to effectively use the textural and frequency features extracted from Gabor convolution, we design a feature extraction module and use the residual module, channel, and spatial attention to further extract features. Simultaneously, we introduce a new diversity loss to encourage the feature extraction module to learn features of different scales and directions. Second, to prevent subtle differences from disappearing in the deep layer, we enhance the textural features obtained from the shallow layer and then fuse the low-level textural features with the textural features obtained by the feature extraction module as the global textural feature representation. Finally, we feed the final feature of the backbone into the attention module and fuse the output with the global textural feature to obtain the final feature.

To demonstrate the effectiveness of our multi-feature fusion network, we conducted extensive experiments on a standard benchmark set, which included FaceForensics++ [8], Celeb-DF [20], and DFD [21]. These show that our method is superior to the binary classifier baselines and achieves state-of-the-art performance. In summary, the contributions of this paper are threefold, as described below:

1. We are the first to design a custom convolution that adaptively learns textural and frequency features for the deepfake detection task with reference to the signal processing method, which brings a novel perspective on the use of textural and frequency features.
2. We propose a new multi-feature fusion network to combine RGB features with textural and frequency features. We also introduce a new diversity loss to encourage the feature extraction module to learn features of different scales and directions.
3. Extensive experiments demonstrate that our method outperforms the binary classification baselines and achieves state-of-the-art detection performance.

The topic in the first section is the introduction. The following sections are structured as follows: Section 2 introduces related work, Section 3 introduces background knowledge, Section 4 introduces the methods we use, Section 5 introduces the experimental results, and Section 6 is the conclusion.

## 2. Related Work

Goodfellow [1] proposed a generative adversarial network (GAN) that had a profound impact on machine learning in 2014, which significantly improved image generation technology. Forged images and videos generated by GANs are widely disseminated on the internet. A generative adversarial network consists of two models: a generator and a classifier. The generator learns the natural data distribution, and the discriminator aims to estimate the probability of the sample having been forged. This process can be transformed into a min-max problem: minimizing generator loss and maximizing discriminator loss. The generator and the discriminator can be regarded as the two sides of a game. The game mode is as follows: The generator generates images -> the discriminator learns how to detect fake images -> the generator is improved and generates new samples -> loop until the generator and the discriminator cannot be improved.

### 2.1. Deepfake Generation Technology

Initially, generative adversarial networks [22,23,24] could only generate low-resolution images. The generation of high-resolution images caused mode collapse, and later GANs gradually solved this problem. Progan [2] proposed a new training method by learning from low resolution and then increasing the resolution, finally learning higher-resolution image generation. Stylegan [3] is an extension of Progan and proposes a new generator structure. It can increase the resolution and control high-level attributes of generated images, such as hairstyles, freckles, etc. However, the AdaIN normalization used in stylegan has droplet artifacts. StyleGAN2 [25] corrects this defect and improves the image quality. Sngan [26] proposes a spectrum normalization technique to stabilize the discriminator training process. Mmdgan [27] combines the idea of generating a moment-matching network and a genetic algorithm.

### 2.2. Deepfake Detection Technology

The initial deepfake detection research mainly used handmade facial features, such as eye color [12], 3D head pose [28], and facial movement [29]. Currently, most of the detection methods use a CNN to extract features for detection. The authors of [30] used specific artifacts, such as color and shape, in the synthesis process for detection. Detection methods based on the spatial domain are strongly correlated with the structure of the generation network and training datasets and lack generalization ability. Recently, methods using frequency features have been proposed. Durall et al. [18] used DFT to extract frequency domain information and to average the amplitudes of different frequency bands. The authors of [19] proposed two frequency domain features, FAD and LFS. The former uses a learnable filter to adaptively decompose the image in the frequency domain and find traces of forgery in different frequency band components. The latter extracts local statistical frequency information and is sensitive to traces of forgery in details. However, the latest deepfake method takes into account the frequency domain defects. The authors of [31] modified the loss function and added a frequency loss term. The authors of [32] performed a shallow reconstruction of fake images by learning a linear dictionary and aimed to reduce the artifacts introduced in the process of image synthesis. Therefore, a frequency-based detection method is not ideal for the newest deepfake dataset. Some studies have also noticed that there are textural differences in fake images [17]. The receptive field of a GAN is limited and cannot capture global textural features, so a texture-based detection network was proposed [33].

In this paper, we first extract and enhance the shallow textural features in RGB images, and then fuse them with the features obtained by the feature extraction module.

## 3. Background

### 3.1. Discrete Cosine Transform

The discrete cosine transform (DCT) is a separable transform, and the transformer core is a cosine function. The DCT has general orthogonal transform properties, and its basis vectors can also describe the relevant characteristics of human speech signals and image signals. DCT conversion is considered to be the best in the conversion of voice signals and image signals.

The two-dimensional DCT change is defined as follows:

(1) $$\begin{array}{c} F(u,v)=\frac{2}{\sqrt{MN}}c\left(u\right)c\left(v\right)\sum_{i=1}^{M-1}\sum_{j=0}^{N-1}f\left(i,j\right)cos\left[\frac{(i+0.5)\pi }{M}u\right]cos\left[\frac{(j+0.5)\pi }{N}v\right] \end{array}$$

(2) $$c\left(x\right)=\left\{\begin{array}{cc} \frac{1}{\sqrt{2}} & x=0\\ 1 & x\neq 0 \end{array}\right.$$

**Figure 2:**
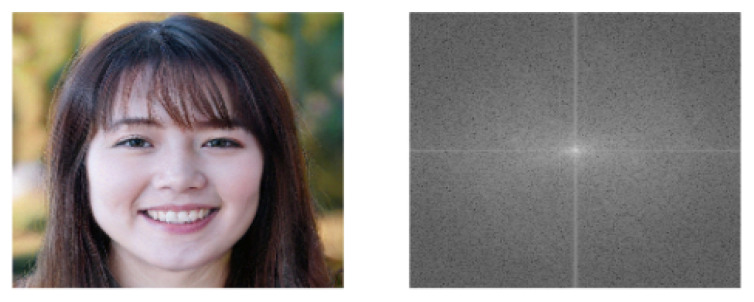
Examples of DCT changes. **Left**: the input image. **Right**: the power spectrum.

In the formula, f(i,j) represents the original signal, F(u,v) is the coefficient after the DCT transformation, M and N represent the number of points of f(i,j), and c(x) is the compensation coefficient to transform the DCT matrix into an orthogonal matrix. The effect of the DCT change is shown in Figure 2.

### 3.2. Frequency Domain Defects

Although GAN models have various structures, most GAN models use the same upsampling modules. Transposed convolution (also known as deconvolution) and nearest-neighbor interpolation are often used in upsampling modules. The upsampling process is as follows: Given a low-resolution feature map as input, the horizontal and vertical resolution are increased by *m* times. For the convenience of explanation, let m=2, add a zero row/column after each row/column of the feature map during the upsampling process, and then apply the convolution operation to re-assign the zero value. Odena et al. [34] found that inserting zeros into a low-resolution image can be considered as copying multiple samples of the original high-frequency spectrum to the generated high-resolution image spectrum. The resulting artifacts are called “checkerboard artifacts”. The latest methods often remove or reduce high-frequency components to prevent such defects. The subsequent convolution kernel uses a low-pass filter, but the low-pass filter cannot completely remove the artifacts. If too much high-frequency content is removed, the final images may become too blurry, making it easy to distinguish them from natural images.

### 3.3. Gabor Filter

A Gabor filter is a linear filter used for edge extraction. The frequency and direction expression of the Gabor filter is similar to that of human eyes, which makes it suitable for textural expression and separation. A two-dimensional Gabor filter is a Gaussian kernel function modulated by a sinusoidal plane wave in the spatial domain.

The mathematical expression of the two-dimensional Gabor function is given below:

Complex:

(3) $$g\left(x,y;\lambda ,\theta ,\psi ,\sigma ,\gamma \right)=exp\left(-\frac{x^{\prime }+\gamma^{2}{y\prime }^{2}}{2\sigma^{2}}\right)exp\left(i\left(2\pi \frac{x^{\prime }}{\lambda }+\psi \right)\right)$$

Real:

(4) $$g\left(x,y;\lambda ,\theta ,\psi ,\sigma ,\gamma \right)=exp\left(-\frac{x^{\prime }+\gamma^{2}{y\prime }^{2}}{2\sigma^{2}}\right)cos\left(2\pi \frac{x^{\prime }}{\lambda }+\psi \right)$$

Imaginary:

(5) $$g\left(x,y;\lambda ,\theta ,\psi ,\sigma ,\gamma \right)=exp\left(-\frac{x^{\prime }+\gamma^{2}{y\prime }^{2}}{2\sigma^{2}}\right)sin\left(2\pi \frac{x^{\prime }}{\lambda }+\psi \right)$$

where

(6) $$x^{\prime }=x\cos\theta +y\sin\theta$$

and

(7) $$y^{\prime }=-x\sin\theta +y\cos\theta$$

The following describes the meaning of each parameter in the formula.

- Wavelength (λ): λ represents the wavelength of the sinusoidal factor. Its value is specified in pixels and is usually not less than 2.
- Direction (θ): θ represents the orientation of the normal to the parallel stripes of a Gabor function.
- Phase shift (ψ): ψ is the maximum offset in the process of modulating the signal.
- Aspect ratio (γ): γ is the spatial aspect ratio and specifies the ellipticity of the support of the Gabor function.
- σ: σ is the sigma/standard deviation of the Gaussian envelope.

For our experiments, we designed a custom Gabor convolution with reference to the Gabor filter. We used real values of the Gabor function as the Gabor convolution kernel function. The aspect ratio was set to 1, and the others were learnable parameters.

## 4. Method

### 4.1. Overview

In this section, we initially state the motivation for the design and give a brief overview of our framework. As mentioned previously, to improve the generalization ability of the model, most current detection methods introduce frequency features or textural features. These methods generally use the spectrum obtained by the discrete Fourier transform as frequency features, directly extract features from RGB images, and consider them to contain textural information. However, the discrete Fourier transform does not match the translation invariance and local consistency of natural images. Therefore, the convolutional network can not be used to extract features, which reduces the classification efficiency of the classification network. In addition, traditional signal processing methods have mature textural and frequency feature extraction technologies. Thus, we argue that using the features obtained by a signal processing method as auxiliary input can be more efficient for collecting textural and frequency features for the deepfake detection task. Meanwhile, the ReLU activation function, which is commonly adopted by current deepfake detection approaches, is replaced with the Swish activation function in our framework. We observed that using the ReLU activation function in the deep convolutional network would lead to a large number of negative gradients being set to zero, thus preventing many neurons from being activated. On the other hand, the slight artifacts caused by forgery methods tend to be preserved in the textural information of shallow features according to [35]. Therefore, more shallow features should be focused on and enhanced.

**Figure 3:**
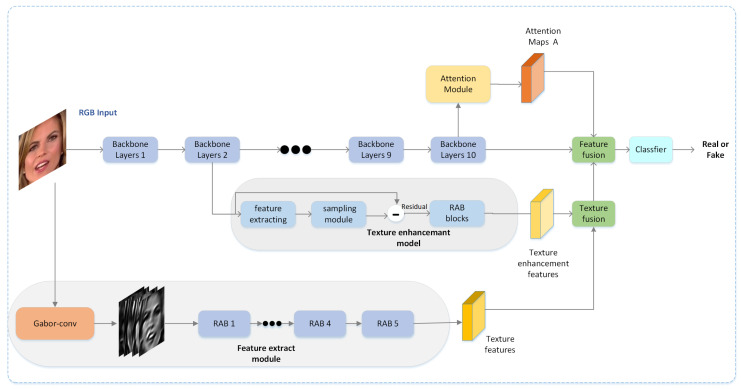
Model architecture: Four components play an essential role in our framework: a feature extraction module to further extract textural and frequency features using a Gabor convolution and residual attention blocks, an attention module for generating attention maps, a texture enhancement block for zooming into the subtle textural information in shallow layers, and two instances of feature fusion for the aggregation of textural, frequency, and semantic features.

Motivated by these observations, we propose a deepfake detection framework fusing RGB, textural, and frequency features. In our framework, four key components are integrated into the backbone network: (1) We employ a feature extraction module to extract textural features and frequency features using a Gabor convolution and residual attention blocks. (2) We use densely connected dilated convolutional layers and residual attention blocks as a texture enhancement block, which can zoom into the subtle textural features in shallow layers. (3) We employ an attention module to generate attention maps. (4) We combine the textural features obtained by the feature extraction module and the shallow enhanced texture and then fuse the final textural features and RGB features. The framework of our method is depicted in Figure 3.

### 4.2. Multi-Feature Fusion Framework

We denote the input face image of the network as *I* and the backbone network of our framework as *f*; the feature maps extracted from the intermediate stage of *t*-th layer are denoted as $f_{t}\left(I\right)$ with the size of $H_{t}\times W_{t}\times C_{t}$. Here, $C_{t}$ is the number of channels, and $H_{t}$ and $W_{t}$ are the height and the width of the feature maps, respectively. The backbone of our framework is xception [36].

#### 4.2.1. Feature Extraction Module

**Figure 4:**
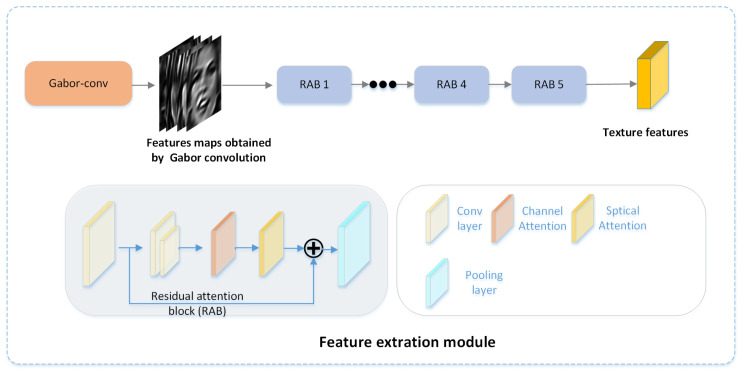
The structure of the feature extraction module. This module is used to extract textural and frequency features from the feature maps obtained by the Gabor convolution.

As described above, given a real/fake face image *I* as input, we first feed RGB images to the Gabor convolution to obtain textural and frequency features at different scales and directions. As shown in Figure 4, the feature extraction module then uses the residual attention blocks to extract features from the feature maps obtained by the Gabor convolution. The residual attention block consists of 3 × 3 convolution layers, pooling layers, channel attention, spatial attention, non-linear activation layers, Swish, and the residual connection. The CBAM structure was adopted for the channel attention and spatial attention [37].

#### 4.2.2. Textural Feature Enhancement

**Figure 5:**
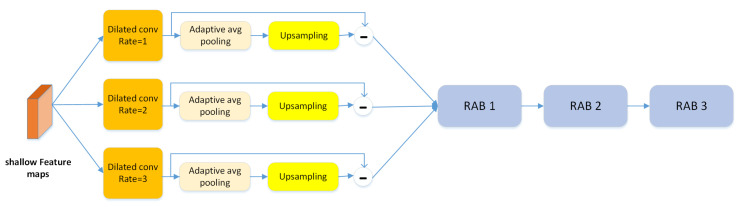
The structure of the texture enhancement module. This module is used to zoom into the subtle textural features in shallow layers.

The artifacts caused by forgery methods are usually salient in the textural information of shallow feature maps. Thus, we design a textural feature enhancement block to preserve more textural information for capturing those artifacts, as shown in Figure 5. We first apply the dilated convolution Dil to obtain feature maps $FL_{t}$ with different granularities from a specific layer $SL_{t}$. Then, we apply adaptive pooling in patches to downsample $FL_{t}$ and obtain the pooled feature map *D*. Finally, we use bilinear interpolation BI to restore *D* to the same size as $DL_{t}$. We define the residual at the feature level with reference to [35] to represent the textural information as follows:

(8) $$T_{SL_{t}}=\sum_{i=1}^{n}\left(FL_{t_{i}}-BI\left(D_{i}\right)\right)$$

(9) $$FL_{t}=Dil\left(f_{SL_{t}}\left(I\right)\right)$$

Here, *T* contains most textural information of $f_{SL_{t}}$. We then use three residual attention blocks to enhance *T*; the output is denoted as $F\in R^{H_{s}\times W_{s}\times C_{F}}$, which is defined as an “enhanced textural feature map”.

#### 4.2.3. Attention Module

**Figure 6:**
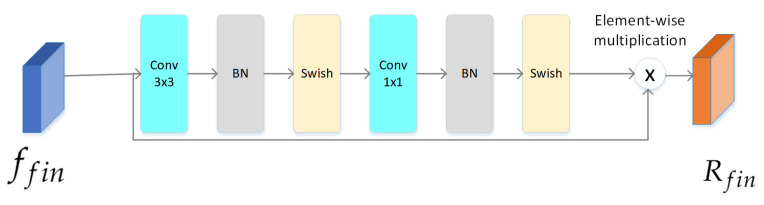
The structure of the attention module. This module is used to generate final RGB feature maps.

Given an image *I* as input, our framework first uses the backbone to generate final feature maps $f_{fin}$ for *I*. Then, we apply the attention module to generate multiple attention maps for $f_{fin}$. As shown in Figure 6, the attention module is a light-weight module that consists of a 3 × 3 convolutional layer, a 1 × 1 convolutional layer, two batch normalization layers, and two non-linear activation layers (Swish). As described above, the ReLU activation function in the deep convolutional network will cause neurons to be unable to be activated. We use the Swish activation function instead of ReLU. The attention module generates attention maps *A* with a size of $H_{t}\times W_{t}$. We multiply $f_{fin}$ and *A* to obtain the final RGB feature maps $R_{fin}$.

(10) $$R_{fin}=f_{fin}\times A$$

### 4.3. Diversity Loss

As described above, Gabor convolution can obtain textural and frequency feature maps of different scales and directions. We use the feature extraction module to process the obtained feature maps. In order to reduce the overlap of the output feature vectors, we propose a cosine-similarity-based regularization term that penalizes feature vectors of the same direction and scale for overlapping with each other.

We first separate the channels $f_{d}$ of the output feature maps, and each channel represents the feature map of a specific direction or scale. Then, the cosine similarity between the channels is calculated. This allows us to penalize the similarity between the feature vectors up to a threshold, leading to more diverse representations. The diversity loss is defined as follows:

(11) $$R_{div}=\sum_{i\neq j}max\left(0,\cos\left(f_{d_{i}},f_{d_{j}}\right)-s_{max}\right)$$

where $s_{max}$ is a hyperparameter for the maximum similarity allowed. For the objective function of our framework, we combine this diversity loss with the traditional cross-entropy loss.

(12) $$L=\lambda_{1}*L_{CE}+\lambda_{2}*R_{div}$$

where $L_{CE}$ is the cross-entropy loss, $R_{div}$ is the diversity loss, $\lambda_{1}$, and $\lambda_{2}$ is the balancing weight for these two terms. By default, we set $\lambda_{1}=\lambda_{2}=1$ in our experiments.

## 5. Experiments

This section mainly describes experiments conducted on deepfake video and image datasets. Section 5.1 provides the experimental details, including the experimental parameters, datasets, and evaluation criteria. Section 5.2 compares the within-dataset performance of this method with mainstream methods. Section 5.3 describes the ablation experiment. Section 5.4 provides an evaluation of the generalization ability. Section 5.5 describes an experiment on robustness to common image disturbances.

### 5.1. Experimental Setup

#### 5.1.1. Datasets

The most challenging deepfake datasets were used in our experiments, including FaceForensics++ [8], Deepfake Detection (DFD) [21], and Celeb-df v2 [20]. FaceForensics++ is a forensics dataset consisting of 1000 original video sequences that have been manipulated with four automated face manipulation methods. The DFD dataset has more than 3000 forged videos from 28 actors with different scenes. The Celeb-DF (v2) dataset contains real and forged videos, and the video quality is similar to that of videos broadcasted online. Celeb-DF includes 590 original videos from YouTube, including different ages, races, and genders. For deepfake images, we used Sngan [26] to generate fake images and used the CelebA [38] datasets as real samples.

#### 5.1.2. Evaluation Standard

We took the accuracy and area under the receiver operating characteristic curve (AUC) as evaluation indicators. The accuracy and AUC are common indicators in deepfake detection tasks.

#### 5.1.3. Experimental Parameters

The environment used in this article was a Linux system. Keras and TensorFlow were used for the model implementation and simulations. The GPU on the server was a Tesla V100, and the memory was 16 GB. The number of epochs was 64, and the batch size was 16. The initial learning rate was 1 × $10^{-4}$, and the weight decay was 1 × $10^{-6}$.

### 5.2. Within-Dataset Experiment

This section compares our method with the previous and state-of-the-art forgery detection methods on the FaceForensics++ [8] dataset. We first evaluated our methods on different video compression settings, including high quality (HQ (c23)) and low quality (LQ (c40)). As shown by the results in Table 1, our method achieved state-of-the-art performance on both settings. It is worth mentioning that our method had a significant improvement in the low-quality setting. Furthermore, we also evaluated our approach on different face manipulation methods in FaceForensics++ [8]. The results are shown in Table 2. We trained and tested our models exactly on low-quality videos for each manipulation method. The results demonstrate that our method outperformed the state-of-the-art methods on all manipulation methods.

### 5.3. Ablation Experiment

To demonstrate the benefit of each module, we evaluated the proposed model on FaceForensics++ [8]. We tested from the backbone and gradually added modules. The first feature fusion was performed while adding the texture extraction and enhancement module. The second feature fusion was performed while adding the attention module.

Table 3 shows the experimental results. The experimental results show that each module of MFF-Net can effectively improve the deepfake detection performance. When the feature extraction and enhancement module were added for the first time, the detection effect was greatly improved. This shows that the fusion of textural and frequency features extracted by the feature extraction and enhancement module and features extracted by the neural network can improve the detection performance.

### 5.4. Generalization Ability Evaluation

Table 4 shows the benchmark results of our framework on the detection of popular unseen deepfake datasets. We evaluated the transferability of our method to DeepfakeDetection [21] and Celeb-DF [20], given that it was trained only on FaceForensics++ (HQ). In the comparative experiment, we compared our method with DPNet [49], SPSL [45], F${}^{3}$-net [19], and Multi-attentional Detection [35]. The generalization ability of our method on Celeb-DF was slightly lower than that of SPSL, which was very strong in the cross-dataset evaluation, but it was higher than those of the other state-of-the-art methods.

### 5.5. Robustness Experiment

This section evaluates the module’s resistance to common image disturbances, including blurring, cropping, compression, adding random noise, and their combinations. We also tested the effect of adversarial training to deal with image disturbances. We retrained MFF-Net on images generated by Sngan [26] with the combined perturbation added. We compared our method with those of [17,51]. The authors of [51] proposed Lip Forensics, a detection approach using high-level semantic irregularities in mouth movements. The authors of [17] proposed Gram-Net, which leverages global textural representations of images for robust detection. Both methods show strong robustness to image disturbances.

#### 5.5.1. Experimental Setup

To create a disturbance dataset, we iterated on all images of the original dataset and applied disturbances with a 50% probability. The created dataset had 50% disturbance data. During the iteration, the combined disturbances were applied in the following order: blur, crop, compression, and noise. The various disturbance settings are described as follows:

- Blur: Filtered by a Gaussian filter with a kernel size randomly sampled from (3, 5, 7, 9);
- Cropping: The picture was randomly cropped along the x- and y-axes. The cropping percentage was sampled from U(5, 20), and the cropped image was resized to the original resolution;
- Compression(JPEG): JPEG compression was applied, and the remaining quality factor was sampled from U(8,80);
- Noise: Inner-diameter Gaussian noise was added to the image. The Gaussian distribution variance was randomly sampled from U(5.0, 20.0).

#### 5.5.2. Experimental Results

The results of the robustness test are shown in Table 5. Our method had better resistance to compression, cropping, noise, and combined disturbances than the other methods. The resistance to blur perturbation was slightly poorer than that of LipForensics [51]. The results also show that adversarial training can effectively improve the robustness, showing the feasibility of confrontation training.

## 6. Conclusions

With this paper, we are the first to combine a signal processing method with a neural network to fuse the textural and frequency features extracted by Gabor convolution with the shallow textural information of RGB images. We propose a multi-feature fusion deepfake detection framework, MFF-Net. The feature extraction module extracts textural and frequency features containing different direction and scale information through Gabor convolution. The texture enhancement module enhances the textural features from the shallow layers to capture more subtle artifacts. Then, the features extracted by the backbone are fed into the attention module to learn discriminative local regions. A diversity loss function is introduced to penalize feature vectors of the same direction and scale for overlapping with each other. A large number of experiments proved that the proposed MFF-Net achieved state-of-the-art performance in deepfake detection and had good performance in detecting unknown datasets; it also had good robustness against common image disturbances. In the future, we intend to introduce an attention mechanism into the feature fusion process to learn the correlations between different modes.

## Figures and Tables

**Table 1:** Quantitative comparison on the FaceForensics++ dataset with the high-quality and low-quality settings. The best performances are marked in bold.

Method	LQ		HQ	
	ACC	AUC	ACC	AUC
Steg.Features [39]	55.98%	-	70.97%	-
LD-CNN [40]	58.69%	-	78.45%	-
Constrained Conv [41]	66.84%	-	82.97%	-
CustomPooling CNN [42]	61.18%	-	79.08%	-
MesoNet [10]	70.47%	-	83.10%	-
Face X-ray [11]	-	61.60%	-	87.40%
Xception [36]	86.86%	89.30%	95.73%	96.30%
Xception-ELA [43]	79.63%	82.90%	93.86%	94.80%
Xception-PAFilters [44]	87.16%	90.20%	-	-
SPSL [45]	81.57%	82.82%	91.50%	95.32%
F${}^{3}$-net [19]	90.43%	93.30%	97.52%	98.10%
Multi-attentional Detection [35]	88.69%	90.40%	97.60%	99.29%
MFF-net	**92.21%**	**95.58%**	**98.18%**	**99.62%**

**Table 2:** Quantitative results (Acc) on the FaceForensics++ (LQ) dataset with four manipulation methods, i.e., DeepFakes (DF) [46], Face2Face (F2F) [47], FaceSwap (FS) [7], and NeuralTextures (NT) [9]. The bold results are the best.

Method	DF	F2F	FS	NT
Steg.Features [39]	67.00%	48.00%	49.00%	56%
LD-CNN [40]	75.00%	56.00%	51.00%	62.00%
Constrained Conv [41]	87.00%	82.00%	74.00%	74.00%
CustomPooling CNN [42]	80.00%	62.00%	59.00%	59.00%
MesoNet [10]	90.00%	83.00%	83.00%	75.00%
Xception [36]	96.01%	93.29%	94.71%	79.14%
Slowfast [48]	97.53%	94.93%	95.01%	82.55%
SPSL [45]	93.48%	86.02%	92.26%	76.78%
F${}^{3}$-net(Xception) [19]	97.97%	95.32%	96.53%	83.32%
F${}^{3}$-net(Slowfast) [19]	98.62%	95.84%	97.23%	86.01%
MFF-Net	**99.73%**	**96.38%**	**98.20%**	**91.79%**

**Table 3:** MFF-Net ablation experiment on FaceForensics++. We verified the effectiveness of the components by adding modules step by step. The evaluation indicators are the ACC and AUC.

Method	LQ		HQ	
	ACC	AUC	ACC	AUC
backbone(xception)	86.86%	89.30%	95.73%	96.30%
+Feature extraction and enchancement module	91.10%	93.39%	97.60%	98.74%
+Attention module	91.32%	94.23%	97.94%	99.15%
+Diversity loss	92.21%	95.58%	98.18%	99.62%

**Table 4:** Generalization ability evaluation on unseen datasets. The evaluation indicator is the AUC. The bold results are the best.

Method	FF++	DFD	Celeb-DF
Xception [36]	96.30%	91.27%	65.50%
ProtoPNet [50]	97.95%	84.46%	69.33%
DPNet [49]	99.20%	92.44%	68.20%
SPSL [45]	96.91%	-	**76.88%**
F${}^{3}$-net [19]	98.10%	-	65.17%
Multi-attentional Detection [35]	**99.80%**	-	67.44%
MFF-Net	99.73%	**92.53%**	75.07%

**Table 5:** Results of the robustness experiment. The training was to use clean datasets of images generated by Sngan and CelebA; the testing was to apply five kinds of perturbations to the test set. Training with the perturbation dataset was used for comparison (the training and testing sets imposed the same disturbances). The evaluation indicator is the AUC. CD: training on clean datasets; PD: training on perturbed datasets.

Method	Train	Test	Blur	Cropping	JPEG	Noise	Combined
Res-Net	Sngan vs. CelebA	Sngan vs. CelebA	82.87%	94.40%	97.12%	87.37%	88.98%
LipForensics [51]	FF++	FF++	96.10%	96.21%	95.60%	73.80%	-
Gram-Net [17]	Stylegan vs. CelebA-HQ	Stylegan vs. CelebA-HQ	94.20%	97.10%	99.05%	92.47%	-
MFF-Net	Sngan vs. CelebA (CD)	Sngan vs. CelebA	94.64%	99.99%	99.98%	98.80%	98.73%
	Sngan vs. CelebA (PD)	Sngan vs. CelebA	97.95%	99.23%	99.38%	98.79%	99.74%

## Data Availability

Data sharing is not applicable for this article.

## References

[1] Goodfellow I., Pouget-Abadie J., Mirza M., Xu B., Warde-Farley D., Ozair S., Courville A., Bengio Y. (2020). Generative adversarial networks. Commun. ACM.

[2] Karras T., Aila T., Laine S., Lehtinen J. Progressive Growing of GANs for Improved Quality, Stability, and Variation. Proceedings of the International Conference on Learning Representations.

[3] Karras T., Laine S., Aila T. A style-based generator architecture for generative adversarial networks. Proceedings of the IEEE/CVF Conference on Computer Vision and Pattern Recognition.

[4] Brock A., Donahue J., Simonyan K. Large Scale GAN Training for High Fidelity Natural Image Synthesis. Proceedings of the International Conference on Learning Representations.

[5] West J., Bergstrom C. (2019). Which Face is Real?. http://www.whichfaceisreal.com.

[6] github FaceAPP. https://faceapp.com/app.

[7] github faceswap. https://github.com/MarekKowalski/FaceSwap/.

[8] Rossler A., Cozzolino D., Verdoliva L., Riess C., Thies J., Nießner M. Faceforensics++: Learning to detect manipulated facial images. Proceedings of the IEEE/CVF International Conference on Computer Vision.

[9] Thies J., Zollhöfer M., Nießner M. (2019). Deferred neural rendering: Image synthesis using neural textures. Acm Trans. Graph. (TOG).

[10] Afchar D., Nozick V., Yamagishi J., Echizen I. Mesonet: A compact facial video forgery detection network. Proceedings of the 2018 IEEE International Workshop on Information Forensics and Security (WIFS).

[11] Li L., Bao J., Zhang T., Yang H., Chen D., Wen F., Guo B. Face X-ray for more general face forgery detection. Proceedings of the IEEE/CVF Conference on Computer Vision and Pattern Recognition.

[12] Matern F., Riess C., Stamminger M. Exploiting visual artifacts to expose deepfakes and face manipulations. Proceedings of the 2019 IEEE Winter Applications of Computer Vision Workshops (WACVW).

[13] Tolosana R., Vera-Rodriguez R., Fierrez J., Morales A., Ortega-Garcia J. (2020). Deepfakes and beyond: A survey of face manipulation and fake detection. Inf. Fusion.

[14] Nguyen H.H., Fang F., Yamagishi J., Echizen I. Multi-task learning for detecting and segmenting manipulated facial images and videos. Proceedings of the 2019 IEEE 10th International Conference on Biometrics Theory, Applications and Systems (BTAS).

[15] Frank J., Eisenhofer T., Schönherr L., Fischer A., Kolossa D., Holz T. Leveraging frequency analysis for deep fake image recognition. Proceedings of the International Conference on Machine Learning.

[16] Zhang X., Karaman S., Chang S.F. Detecting and simulating artifacts in gan fake images. Proceedings of the 2019 IEEE International Workshop on Information Forensics and Security (WIFS).

[17] Liu Z., Qi X., Torr P.H. Global texture enhancement for fake face detection in the wild. Proceedings of the IEEE/CVF Conference on Computer Vision and Pattern Recognition.

[18] Durall R., Keuper M., Pfreundt F.J., Keuper J. (2019). Unmasking deepfakes with simple features. arXiv.

[19] Qian Y., Yin G., Sheng L., Chen Z., Shao J. Thinking in frequency: Face forgery detection by mining frequency-aware clues. Proceedings of the European Conference on Computer Vision.

[20] Li Y., Yang X., Sun P., Qi H., Lyu S. Celeb-df: A large-scale challenging dataset for deepfake forensics. Proceedings of the IEEE/CVF Conference on Computer Vision and Pattern Recognition.

[21] Deepfakedetection. https://ai.googleblog.com/2019/09/contributing-data-to-deepfake-detection.html.

[22] Arjovsky M., Chintala S., Bottou L. Wasserstein generative adversarial networks. Proceedings of the International conference on machine learning.

[23] Berthelot D., Schumm T., Metz L. (2017). Began: Boundary equilibrium generative adversarial networks. arXiv.

[24] Kodali N., Abernethy J., Hays J., Kira Z. (2017). On convergence and stability of gans. arXiv.

[25] Karras T., Laine S., Aittala M., Hellsten J., Lehtinen J., Aila T. Analyzing and improving the image quality of stylegan. Proceedings of the IEEE/CVF Conference on Computer Vision and Pattern Recognition.

[26] Miyato T., Kataoka T., Koyama M., Yoshida Y. Spectral Normalization for Generative Adversarial Networks. Proceedings of the International Conference on Learning Representations.

[27] Li C.L., Chang W.C., Cheng Y., Yang Y., Póczos B. MMD GAN: Towards deeper understanding of moment matching network. Proceedings of the 31st International Conference on Neural Information Processing Systems.

[28] Yang X., Li Y., Lyu S. Exposing deep fakes using inconsistent head poses. Proceedings of the ICASSP 2019-2019 IEEE International Conference on Acoustics, Speech and Signal Processing (ICASSP).

[29] Agarwal S., Farid H., Gu Y., He M., Nagano K., Li H. Protecting World Leaders Against Deep Fakes. Proceedings of the CVPR Workshops.

[30] Carvalho T., Faria F.A., Pedrini H., Torres R.d.S., Rocha A. (2015). Illuminant-based transformed spaces for image forensics. IEEE Trans. Inf. Forensics Secur..

[31] Durall R., Keuper M., Keuper J. Watch your up-convolution: Cnn based generative deep neural networks are failing to reproduce spectral distributions. Proceedings of the IEEE/CVF Conference on Computer Vision and Pattern Recognition.

[32] Huang Y., Juefei-Xu F., Wang R., Guo Q., Ma L., Xie X., Li J., Miao W., Liu Y., Pu G. Fakepolisher: Making deepfakes more detection-evasive by shallow reconstruction. Proceedings of the 28th ACM International Conference on Multimedia.

[33] Geirhos R., Rubisch P., Michaelis C., Bethge M., Wichmann F.A., Brendel W. ImageNet-trained CNNs are biased towards texture; increasing shape bias improves accuracy and robustness. Proceedings of the International Conference on Learning Representations.

[34] Odena A., Dumoulin V., Olah C. (2016). Deconvolution and checkerboard artifacts. Distill.

[35] Zhao H., Zhou W., Chen D., Wei T., Zhang W., Yu N. Multi-attentional deepfake detection. Proceedings of the IEEE/CVF Conference on Computer Vision and Pattern Recognition.

[36] Chollet F. Xception: Deep learning with depthwise separable convolutions. Proceedings of the IEEE Conference on Computer Vision and Pattern Recognition.

[37] Woo S., Park J., Lee J.Y., Kweon I.S. Cbam: Convolutional block attention module. Proceedings of the European conference on computer vision (ECCV).

[38] Liu Z., Luo P., Wang X., Tang X. Deep learning face attributes in the wild. Proceedings of the IEEE international conference on computer vision.

[39] Fridrich J., Kodovsky J. (2012). Rich models for steganalysis of digital images. IEEE Trans. Inf. Forensics Secur..

[40] Cozzolino D., Poggi G., Verdoliva L. Recasting residual-based local descriptors as convolutional neural networks: An application to image forgery detection. Proceedings of the 5th ACM Workshop on Information Hiding and Multimedia Security.

[41] Bayar B., Stamm M.C. A deep learning approach to universal image manipulation detection using a new convolutional layer. Proceedings of the 4th ACM Workshop on Information Hiding and Multimedia Security.

[42] Rahmouni N., Nozick V., Yamagishi J., Echizen I. Distinguishing computer graphics from natural images using convolution neural networks. Proceedings of the 2017 IEEE Workshop on Information Forensics and Security (WIFS).

[43] Gunawan T.S., Hanafiah S.A.M., Kartiwi M., Ismail N., Za’bah N.F., Nordin A.N. (2017). Development of photo forensics algorithm by detecting photoshop manipulation using error level analysis. Indones. J. Electr. Eng. Comput. Sci..

[44] Chen M., Sedighi V., Boroumand M., Fridrich J. JPEG-phase-aware convolutional neural network for steganalysis of JPEG images. Proceedings of the 5th ACM Workshop on Information Hiding and Multimedia Security.

[45] Liu H., Li X., Zhou W., Chen Y., He Y., Xue H., Zhang W., Yu N. Spatial-phase shallow learning: Rethinking face forgery detection in frequency domain. Proceedings of the IEEE/CVF Conference on Computer Vision and Pattern Recognition.

[46] github Deepfakes. https://github.com/deepfakes/faceswap.

[47] Thies J., Zollhofer M., Stamminger M., Theobalt C., Nießner M. Face2face: Real-time face capture and reenactment of rgb videos. Proceedings of the IEEE Conference on Computer Vision and Pattern Recognition.

[48] Feichtenhofer C., Fan H., Malik J., He K. Slowfast networks for video recognition. Proceedings of the IEEE/CVF International Conference on Computer Vision.

[49] Trinh L., Tsang M., Rambhatla S., Liu Y. Interpretable and trustworthy deepfake detection via dynamic prototypes. Proceedings of the IEEE/CVF Winter Conference on Applications of Computer Vision.

[50] Chen C., Li O., Tao D., Barnett A., Rudin C., Su J.K. (2019). This looks like that: Deep learning for interpretable image recognition. Adv. Neural Inf. Process. Syst..

[51] Haliassos A., Vougioukas K., Petridis S., Pantic M. Lips Don’t Lie: A Generalisable and Robust Approach To Face Forgery Detection. Proceedings of the IEEE/CVF Conference on Computer Vision and Pattern Recognition.

